# Lead biosorption from industrial wastewater using *Cladophora*

**DOI:** 10.1038/s41598-025-29920-4

**Published:** 2025-12-11

**Authors:** Alaa M. Metwally, Samia A. A. Aly, Sarah O. Makled, Amr M. S. Abdelkader

**Affiliations:** 1 Sanitary Engineering Department, Faculty of Engineering, Alexandria University, Alexandria, Egypt; 2 Oceanography Department, Faculty of Science, Alexandria University, Alexandria, Egypt

**Keywords:** Cladophora glomerata, Silicon dioxide nanoparticles, Lead adsorption, Industrial wastewater, Environmental sciences, Engineering

## Abstract

Discharge of wastewater containing lead ions poses serious risks to plants, aquatic organisms, and human health. Conventional heavy metal removal methods, such as membrane filtration and activated carbon adsorption, are often costly and less effective at trace concentrations. This study investigates the biosorption potential of three adsorbents: the green alga *Cladophora glomerata* (CGM) collected from the Red Sea (Egypt), silicon dioxide (SiO_2_) nanoparticles, and a hybrid composite of CGM with SiO_2_ nanoparticles, for lead removal from industrial wastewater. The adsorbents were characterized using SEM and FTIR, and the effects of pH, adsorbent dose, and other operating parameters (temperature: 20 ± 2 °C, stirring speed: 300 rpm, contact time: 60 min, and initial lead concentration: 20 mgL^− 1^) were investigated. CGM achieved 100% removal efficiency at pH 5.0 with a biomass dose of 1.3 g/L, while SiO_2_ nanoparticles achieved the same efficiency at 0.5 g/L. The hybrid composite reached 97.79% efficiency at 0.7 g/L, suggesting possible competition for adsorption sites. Kinetic studies indicated that the biosorption followed the pseudo-second-order non-linear model with high correlation coefficients (R² = 0.998, 0.998, and 0.9998 for CGM, SiO_2_, and the hybrid, respectively). These findings highlight the strong potential of algae-based, nanoparticle-based, and hybrid biosorbents as cost-effective and environmentally friendly solutions for surface water treatment and wastewater post-treatment.

**Supplementary Information:**

The online version contains supplementary material available at 10.1038/s41598-025-29920-4.

## Introduction

The presence of heavy metal ions in aquatic systems is a worldwide concern. Even at low concentrations, these heavy metals can pose significant risks to public health due to their toxicity, carcinogenic effects, anemia, vomiting, kidney damage, high blood pressure^1^, and inability to biodegrade. Lead ions, one of the most toxic heavy metals, enter the environment by discharging various industrial activities, including storage batteries, pigments, mining, painting, coating, electroplating, insecticides, petrochemicals, and photographic materials^2^. When absorbed into the body, it can cause kidney damage, high blood pressure in adults, and severe effects on the brain and nerves^3^.

Lead ion contamination in wastewater is a significant concern across various industries, with varying concentrations. In battery manufacturing and recycling, lead ion levels can range from 1.0 to 10.0 mgL^− 1^, potentially reaching up to 50.0 mgL^− 1^ in untreated effluents. Other industries, such as metal plating and finishing, report lead concentrations of 0.5 to 5.0 mgL^− 1^, while mining and smelting can vary from 0.1 to 20.0 mgL^− 1^. Paint and pigment manufacturing typically shows levels between 0.5 and 15.0 mgL^− 1^, and electronics manufacturing ranges from 0.1 to 3.0 mgL^− 1^. Additional sources include glass and ceramics production (0.2 to 5.0 mgL^− 1^), textile and dyeing industries (0.1 to 2.0 mgL^− 1^), petroleum refining (0.05 to 2.0 mgL^− 1^), automotive and aerospace industries (0.1 to 4.0 mgL^− 1^), chemical manufacturing (0.5 to 8.0 mgL^− 1^), and pesticide and fertilizer production (0.1 to 5.0 mgL^− 1^). These varying concentrations underscore the necessity for effective wastewater treatment strategies to address lead ion pollution from industrial activities.

To ensure the safety of drinking water, the Environmental Protection Agency (EPA) has set a permissible level for lead at 0.05 mg L^− 1^^4^. Due to the detrimental effects of lead ions and the urgency to reduce their presence in water, there is a significant focus on developing cost-effective and environmentally friendly techniques for treating and eliminating this hazardous pollutant^5^.

The conventional methods used for lead ion reduction in aqueous matrices primarily include precipitation, ion exchange, membrane processes, adsorption with activated carbon, and oxidation and reduction^6^. These techniques are commonly employed to remove lead ions from water and ensure its safety for consumption. Nevertheless, these methods have certain drawbacks, including their high cost, high energy consumption, and inefficiency, particularly when dealing with low concentrations of existing heavy metals.

Adsorption presents a practical and effective solution for eliminating heavy metals, offering advantages such as cost-effectiveness, high efficiency, minimal generation of secondary waste, and environmental friendliness.

By harnessing the natural adsorption capabilities of green algae, the reliance on chemical usage in the waste treatment process can be significantly reduced. This reduction in chemical usage indirectly contributes to lowering the overall cost of the treatment plant. Green algae can be found in various regions worldwide, ranging from airborne or subaerial environments to terrestrial or aquatic habitats, including freshwater and marine ecosystems^7^.

Several studies on microalgae for biosorption applications have been conducted. A study conducted by Farhan (2022) used Chara algae (C. vulgaris) to remove copper and lead ions from aqueous solutions. The results showed that the metal adsorption process takes place quickly at pH values (5.0–6.0) and temperature levels (25–30 °C); The adsorption process is fast and occurs by 90% within the first 15 min^7^.

Another study^8^ used a batch study to investigate the efficiency of Ulva lactuca carbon for lead adsorption from aqueous solution. The results showed that the most beneficial removal was achieved at pH 3.0 and the maximum lead ions absorption of 3.49 mgg-1 ^9^.

A study aimed at finding natural biosorbents that can remove highly hazardous lead ions from aqueous solutions. Citrus limetta peel powder, Banana peel powder, and Betel leaf powder were investigated to absorb synthetic lead ions from an aqueous solution. The most adsorption occurs at pH 6.0 for Banana peel and pH 7.0 for Citrus limetta peel and betel leaf. The percentage removal of lead is attained within 100 min for Citrus limetta peel powder and Betel Leaf, while it takes about 140 min for Banana peel powder.

Algae are inexpensive, can grow in various environments, and are renewable and green, efficiently binding with a wide range of heavy metals, including lead ions. Algae are biodegradable, posing less of a waste disposal problem compared to non-biodegradable synthetic adsorbents like activated carbon. Their disposal is generally more eco-friendly. Algae do not require extensive energy input for production, regeneration, or operation as an adsorbent. Cultivating algae typically relies on sunlight and basic nutrients, which significantly reduces the energy footprint of the process; they pose less risk to humans and the environment during the adsorption and disposal processes^10^.

Most microalgae have polysaccharides, proteins, and lipids on the surface of their cell walls, which are surrounded by a porous three-dimensional macromolecular network^11^. These structures, which have carboxylic, hydroxyl, phosphate, and sulfate groups, help algae adsorb the metals^12^. Carboxyl groups (-COOH) negatively charged sites electrostatically attract Pb^2+^ ions, forming stable complexes through ion exchange or coordination bonding. Hydroxyl groups (-OH) can also participate in metal binding through hydrogen bonding or complexation, enhancing Pb^2+^ adsorption. These functional groups facilitate efficient lead ion capture via electrostatic attraction, complexation, and ion exchange mechanisms^13^.

In recent times, nanometer solid materials have emerged as highly effective solid supports and efficient sorbents for the removal of heavy metals^14^. This is primarily due to their unique properties that make them well-suited for this purpose. An important property of nanometer solid materials is that many atoms with high chemical activity and adsorption capacities are located on the surface of the nanoparticles^15^.

The metal adsorption capacity of microalgae biomass has been proven in studies in the literature^16^. Nanomaterial adsorbents have become a topic of great interest owing to their exceptional properties, such as high adsorption strength, greater surface area, and chemical stability^17^. Within these limited studies on metal adsorption given in the literature, Eco-friendly adsorption processes using nanoparticles or algae are emerging as potential alternatives for heavy metal removal^18^. These processes offer advantages such as low cost, selectivity for specific metals, and short operating time. There is a growing interest in developing highly efficient adsorbents that can match the performance of commercially available adsorbents for the treatment of industrial wastewater.

Algae can be modified to enhance their adsorption capacity and selectivity. Algae offer high biosorption capacity due to their polysaccharide-rich cell walls^19^. Nanomaterials provide a high surface area and chemical stability^20^. Their combination is hypothesized to leverage these complementary properties for superior lead ion removal. A hybrid approach combining microalgae and nanoparticles enhances lead removal by addressing macroalgae’s slow uptake, high nanoparticle synthesis costs, and separation challenges.

This study aims to explore natural and hybrid biosorbents for the efficient removal of hazardous Pb^2+^ ions from aqueous systems. The biosorption potential of *Cladophora glomerata* biomass (CGM), silicon dioxide nanoparticles (SiO_2_NPs), and their composite material was systematically investigated to assess their ability to reduce Pb^2+^ concentrations to environmentally safe levels. Key operational parameters, including solution pH and sorbent dosage, were optimized to enhance removal efficiency and provide insight into the adsorption mechanism. The findings of this work contribute to the development of cost-effective and sustainable strategies for mitigating lead contamination in industrial wastewater.

Unlike conventional biosorption studies that focus on single biomass or inorganic adsorbents, the present work introduces a hybrid biosorbent (CGM–SiO_2_NPs) integrating the natural functional groups of *Cladophora glomerata* with the high surface area of silica nanoparticles. This combination is expected to enhance adsorption capacity and reduce mass transfer limitations, representing a novel and sustainable approach for Pb^2+^ removal from real industrial wastewater.

## Materials and methods

### Collection and Preparation of *Cladophora glomerata* biomass

CGM samples were gathered from various locations around the Red Sea coast of Egypt and manually collected from rocks and reefs during low tide. Along the shoreline, collection is usually done by hand or with nets, especially in September and during peak growth seasons. After rinsing the samples with distilled water to remove salt, sand, and epiphytes, they were air-dried at room temperature, then oven-dried at 60 °C for 24 h to ensure complete drying. The dried samples were ground into a fine powder, sieved to a particle size of 150 μm (100-mesh sieve), and stored in securely sealed dark vials at room temperature until analysis.

### Molecular identification of algal biomass

The algae sample was identified using an 18 S rRNA molecular marker^21^. DNA was purified using the NanoMag Plant and Algae DNA Isolation Kit. The purified DNA was amplified with specific primers using DreamTaq master mix, resulting in an ≈ 869 bp amplicon. PCR products were separated by electrophoresis on a 1.5% agarose gel and visualized under UV light. Purification was carried out with the Gene JET PCR Purification Kit. Data analysis was performed using the Geldoc-it system and Totallab software. Positive amplicons were sequenced using the ABI PRISM Genetic Analyzer, confirming 94.7% similarity to *C. glomerata* as shown in Table 1.

**Table 1:** Molecular identification of the marine Alga sample based on 18 S rRNA gene sequencing.

GenBank Accession No	Closest matching isolate (*C. glomerata*)	Identity (%)

### Preparation of the novel biosorbent (CGM + SiO_2_NPs)

#### SEM before the adsorption of lead

**Fig. 1:**
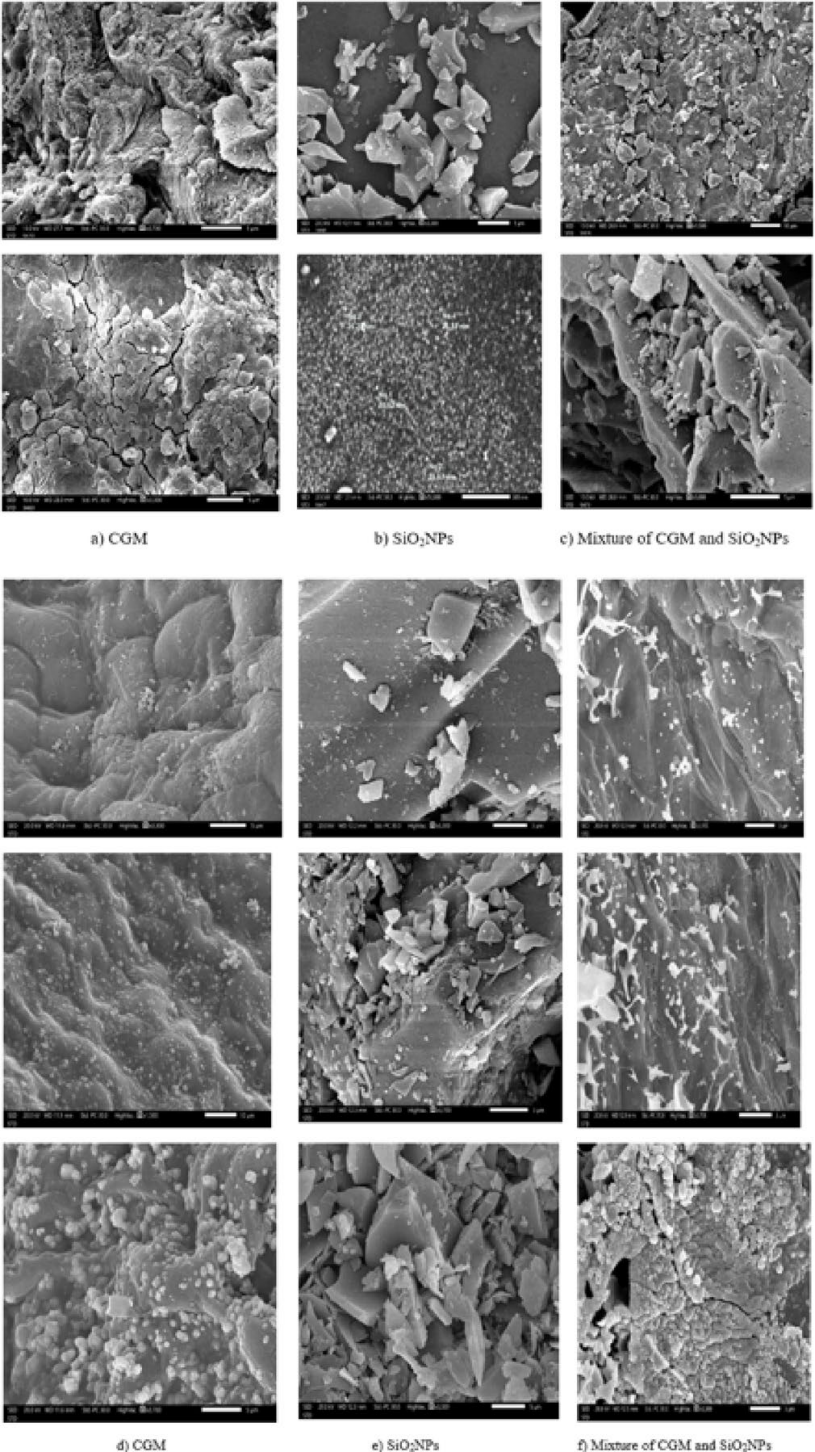
SEM images of **a** C. glomerata **b** Silicon dioxide nanoparticles **c** C. glomerata hybrid with Silicon dioxide nanoparticles before adsorption. SEM images of **d** C. glomerata **e** Silicon dioxide nanoparticles **f** C. glomerata hybrid with Silicon dioxide nanoparticles after adsorption.

The commercially available silicon dioxide nanoparticles (SiO_2_NPs) with a purity of 99.9% were used in this study. The size distribution analysis revealed that SiO_2_NPs were aggregated, with evidence of significant particle clustering (Fig. 1). They have a high surface area-to-volume ratio, which enhances their reactivity and physical properties. They form a novel biosorbent when mixed with CGM in a 1:1 ratio.

Figure 1**(a–c)** shows the surface morphology of CGM, SiO_2_NPs, and their hybrid composite before Pb^2+^ adsorption.

Figure 1a presents a scanning electron micrograph of *Cladophora glomerata* biomass (CGM) at magnifications of ×3,700 and ×3,300. The surface appears rough, porous, and multilayered features that are crucial for adsorption processes. The particle framework of the green microalgae is uneven, with a wrinkled and fractured outer layer that provides an extensive external surface area and numerous active sites for metal ion binding^22^.

Figure 1b displays SEM images of SiO_2_ nanoparticles at magnifications of ×3,300 and ×50,000. The particles exhibit quasi-spherical shapes with diameters in the range of 20–27 nm, confirming their nanoscale nature. These nanosized particles possess a high surface-area-to-volume ratio, which promotes faster adsorption kinetics, greater active site availability, and improved selectivity for heavy metal ions.

 In Fig. 1c, the SEM micrographs of the hybrid composite (CGM–SiO_2_NPs), magnified ×1,500 and ×5,000, reveal a more heterogeneous and interconnected structure. The SiO_2_ nanoparticles are well-dispersed and anchored onto the algal matrix, creating deep pores and channels that increase the effective surface area. This integration enhances mass transfer and allows for more efficient interaction between Pb^2+^ ions and the active functional sites on the hybrid surface. Such synergistic morphological features have been reported to enhance adsorption performance in hybrid bio-nanomaterial systems^23^, ^24^.

#### SEM after the adsorption of lead

The surface morphology of the biosorbents after Pb^2+^ adsorption was examined using Scanning Electron Microscopy (SEM) to visualize the structural changes and confirm metal uptake efficiency (Fig. 1d–f).

For *Cladophora glomerata* (CGM), the micrographs at different magnifications reveal significant surface transformation compared with the pristine biomass. The initially porous and fibrous structure became denser, with roughened regions and numerous granular deposits visibly covering the surface. These bright, spherical particles correspond to Pb^2+^ ions successfully attached to the biomass through ion exchange, complexation, and electrostatic interactions with oxygen- and nitrogen-containing functional groups. The reduction in pore visibility and the formation of compact aggregates indicate effective occupation of active binding sites and confirm the strong affinity of CGM for Pb^2+^ ions.

The SEM images of SiO_2_ nanoparticles (SiO_2__2__2_NPs) after adsorption (Fig. 1e) also display pronounced morphological changes. Compared with the smooth surface of pristine SiO_2_, the Pb^2+^-loaded nanoparticles exhibit rough, heterogeneous surfaces with distinct clusters and agglomerated layers. These features verify the successful deposition of lead ions onto the available silanol (≡ Si–OH) groups through surface complexation and electrostatic attraction. The aggregation of particles and the disappearance of smooth areas confirm that the nanoparticle surface reached saturation, reflecting the high reactivity and surface-area-to-volume ratio characteristic of SiO_2_-based adsorbents. Similar post-adsorption morphological alterations have been reported for silica-based nanomaterials used in heavy metal remediation.

For the hybrid composite (CGM–SiO_2_NPs) (Fig. 1f), the SEM micrographs reveal a distinctly modified surface compared with both individual components. The hybrid surface exhibits a highly heterogeneous structure, with SiO_2_ nanoparticles uniformly distributed within the algal matrix, forming interconnected clusters and porous networks. Following Pb^2+^ uptake, the surface becomes densely covered with granular deposits and compact layers, suggesting extensive metal binding across both organic and inorganic components. The visible pore blockage and the formation of aggregated Pb-rich domains confirm strong Pb^2+^ adsorption and chemisorptive interaction between Pb ions and the composite’s active functional sites.

These morphological observations clearly demonstrate the synergistic enhancement achieved by combining CGM with SiO_2_NPs. The hybrid structure provides improved surface roughness, higher porosity, and greater accessibility of adsorption sites, which collectively reduce mass-transfer resistance and promote rapid metal uptake. Overall, the SEM evidence confirms that all three materials effectively captured Pb^2+^ ions, with the CGM–SiO_2_NPs composite exhibiting the most pronounced structural modification and superior adsorption performance. These findings align with previously reported results for hybrid bio-nanocomposites designed for heavy metal removal^25,26^.

#### Comparative morphological interpretation before and after biosorption

A comparative SEM examination before and after Pb^2+^ adsorption clearly confirms the adsorption mechanism for all tested sorbents. The raw CGM exhibited a rough and porous fibrous structure that facilitates ion diffusion and metal binding; after adsorption, noticeable particle deposition and agglomeration on the surface were observed, indicating the occupation of these pores by Pb^2+^ ions. Similar morphology-driven biosorption behavior has been previously reported for algal biomass. For SiO_2__2_NPs, nanoscale spherical particles with partial aggregation were observed initially, whereas post-adsorption images revealed increased surface roughness and deposited agglomerations, demonstrating successful Pb^2+^ binding. These results correspond with earlier studies where silica-based nanomaterials showed roughened surfaces after heavy-metal adsorption. For the CGM–SiO_2__2_NPs hybrid material, the incorporation of nanoparticles into the algal matrix enhanced surface heterogeneity and pore accessibility before adsorption. After Pb^2+^ uptake, compact aggregation layers were observed, confirming multiple active-site interactions and improved metal fixation. This synergistic performance of hybrid biosorbents is consistent with the reported enhancements in nanocomposite-based adsorption systems^27^.

Overall, these morphological transitions correlate well with the increased removal efficiency recorded experimentally, confirming that the hybrid material benefits from enhanced active sites and reduced mass-transfer resistance.

### Preparation of lead solution

A stock solution of Pb^2+^ (1000 mg L^− 1^) was prepared by dissolving 1.599 g of Pb(NO₃)_2_ in a minimum amount of 1 + 1 HNO₃, and then diluting to a final volume of 1.0 L with reagent-grade water in a volumetric flask. Working solutions with a concentration of 20.0 mg L^− 1^ were subsequently prepared by appropriate dilution of the stock solution, as calculated using Eq. (1). The pH of the working solutions was adjusted to the desired value by the dropwise addition of 0.1 M HCl or 0.1 M NaOH.

1 $$\:Concentration=\frac{Weight\:\left(mg\right)\:x\:gravimetric\:factor}{Volume\:\left(L\right)}\:$$

where: gravimetric factor = the weight fraction of the analyte in the compound.

The pH of each working solution was adjusted to the desired value (2.0–8.0) by the dropwise addition of 1.0 M HCl or 1.0 M NaOH, followed by thorough mixing. No buffer solutions were used, as pH control was achieved solely by acid/base adjustment.

### Analytical and statistical analysis

Samples were collected in dry and clean glass bottles, and all sampling and experiments were performed according to the 20th edition of *Standard Methods for the Examination of Water and Wastewater*. Lead ion concentration was determined using an ICP-OES system (ICAP PRO, Thermo Fisher Scientific, Bremen, Germany) with a limit of detection (LOD) of 0.006 mgL^− 1^ and R² = 1.000. Solution pH was measured using a bench pH meter (AD1000 Professional, Adwa Instruments, Szeged, Hungary) with RS232/USB interface and GLP compliance. Biomass weight was determined using an analytical balance (PRECISE 205 A SuperBal, Precisa Gravimetrics AG, Dietikon, Switzerland) with an accuracy of ± 0.0001 g. Statistical analysis was performed using SPSS software^28^. (version 18.0, IBM Corp., Armonk, NY, USA), and standard deviation was calculated using Microsoft Excel 2019 (Microsoft Corporation, Redmond, WA, USA).

Taking into consideration the determined concentrations, the treatment efficiencies were calculated using Eq. 2. The sorption capacity was calculated from a metal mass balance, as shown in Eq. 3.

2 $$\:\text{R}\:\text{\%}=\frac{(\text{C}\text{o}-\text{C})}{\text{C}\text{o}}\text{x}100$$

where C0 and C (mg $\:{\text{L}}^{-1}$ ) are the initial and residual lead ion concentrations

3 $$\:\text{q}\text{e}=\frac{\left(\text{C}\text{o}-\text{C}\right)\text{V}}{\text{m}}$$

where C0 and C (mg $\:{L}^{-1}$ ) are the initial and residual lead ion concentrations, V (l) is the aqueous volume of the sorption reaction, m (g) is the mass of dry sorbent, and qe (mg g^− 1^) is the sorption metal capacity that represents the amount of the lead ions adsorbed per gram of dry sorbent.

The pseudo-first-order model assumes that the change in lead concentration over time is directly proportional to its first power. It is further based on the assumption that the reaction rate, r, can be expressed as:

4 $$\:-r\:=-\frac{dc\:}{dt}={K}_{1}C$$

Separating and integrating Eq. (4) with respect to the limits *C* = *C*_o_ at *t* = 0 and *C* = *C* at any *t* yields.

$$- \int_{{c_{o} }}^{c} {\frac{{dc}}{c}} = K_{1} \int_{0}^{t} {dt}$$

$$Or~\ln \frac{C}{{C_{o} }} = - K_{1} t$$

The rate constant K_1_​ can be determined from the slope of the plot of ln *(C/C0)* versus *t*. Additionally, the sorption kinetics were further evaluated using a pseudo-second-order model, which is based on the assumption that the following reaction occurs:

$2A{\text{ }} \to {\text{ }}products$ where A represents the dye component accumulating on the solid adsorbent, the reaction rate (r) can be expressed as:

5 $$\:-\text{r}\:=\:-\:\frac{dc}{dt}={K}_{2}{C}^{2}$$

By integrating Eq. (5) within the limits C = C_o_ at time t = 0 and C = C at any time t, the equation simplifies to:

$$\frac{1}{C} = \frac{1}{{C_{o} }} + K_{2} t$$

The data was fitted to a pseudo-second-order model using a Nonlinear curve fitting tool in Origin Pro. The pseudo-second-order model can be expressed as a Non-linear form shown in Eq. 6

6 $$\:qt=\frac{q{e}^{2}x\:K2\:x\:t}{1+K2x\:qe\:x\:t}$$

where “t” represents the contact time in minutes, “qe” (mg g^–1^) refers to the amount of lead ions adsorbed at equilibrium, while “qt” (mg g^– 1^) represents the amount of lead ions adsorbed at any given time, “t”. In this equation, qe represents the equilibrium adsorption capacity, K_2_ is the rate constant, and t is the contact time.

### Fourier transform infrared spectroscopy

#### FTIR before the lead adsorption

**Fig. 2:**
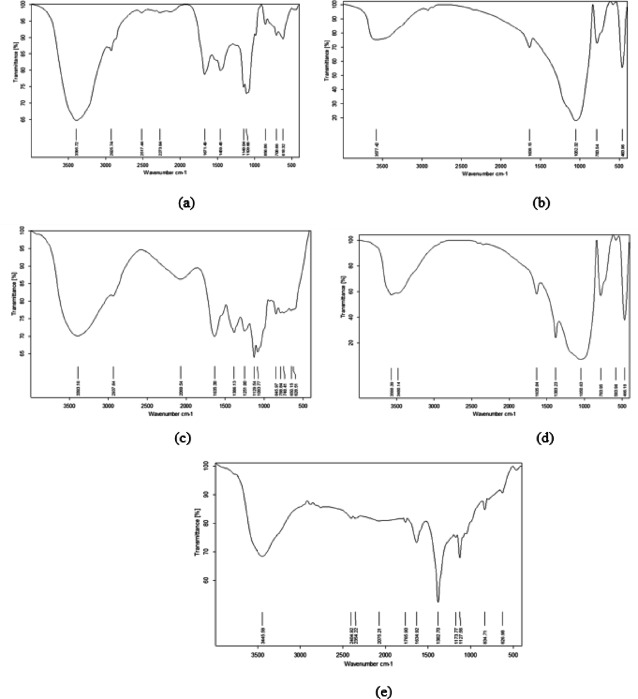
FTIR peaks of transmittance of lead before biosorption **a** CGM and **b** SiO_2_NPs. FTIR peaks of transmittance of lead after biosorption **c** CGM and **d** SiO_2_NPs **e** CGM&SiO2NPs.

The FTIR spectra of *Cladophora glomerata* biomass (CGM) and SiO_2_ nanoparticles before Pb^2+^ adsorption is presented in Fig. 2(a, b). The spectrum of CGM shows several characteristic absorption bands corresponding to major functional groups responsible for metal ion binding. The broad band at 3395 cm^− 1^ is attributed to O–H stretching vibrations of hydroxyl groups, which are abundant in polysaccharides and proteins present in algal cell walls. The peak at 2925 cm^− 1^ corresponds to aliphatic C–H stretching of lipid components, while the small band at 2517 cm^− 1^ is assigned to S–H stretching of thiol groups, indicating sulfur-containing biomolecules. The absorption at 1671 cm^− 1^ represents the C = O stretching of the amide I band from peptide linkages, and the band at 1459 cm^− 1^ corresponds to C–H bending vibrations in lignocellulosic structures. The peak observed at 1149 cm^− 1^ is attributed to C–O–C stretching of polysaccharides, and those at 856 cm^− 1^ and 708 cm^− 1^ correspond to C–H bending in complex organic structures. The small peak at 618 cm^− 1^ is related to C–Cl stretching of alkyl halides.

For SiO_2_ nanoparticles (Fig. 2b), the FTIR spectrum shows a broad band at 3577 cm^− 1^ corresponding to surface hydroxyl (–OH) groups, and a peak at 1638 cm^− 1^ due to H–O–H bending vibrations of adsorbed water molecules, which confirms the hydrophilic nature and high surface area of SiO_2__2_. The strong absorption band at 1052 cm^− 1^ is assigned to asymmetric Si–O–Si stretching, characteristic of the siloxane framework, whereas the peaks at 783 cm^− 1^ and 463 cm^− 1^ correspond to Si–O–Si bending and Si–O deformation, respectively, confirming the well-formed silica network.

These functional groups (hydroxyl, carboxyl, amide, and silanol) play crucial roles as active binding sites for Pb^2+^ ions during biosorption. The presence of such groups indicates that both CGM and SiO_2_NPs have high potential for interaction with metal ions through electrostatic attraction, hydrogen bonding, and surface complexation, as previously reported by^29^ and ^30^. The spectral results also reveal potential chemical compatibility between SiO_2_NPs and the functional moieties of CGM, suggesting the stability of the composite biosorbent and its effectiveness for metal ion removal.

#### FTIR after the lead adsorption

The FTIR spectra of CGM, SiO_2_NPs, and their hybrid composite after Pb^2+^ adsorption is presented in Fig. 2(c–e). Noticeable spectral shifts and intensity changes were observed compared to the pristine materials, confirming the involvement of surface functional groups in the biosorption process.

For CGM (Fig. 2c), the broad band around 3393 cm^− 1^, attributed to O–H and N–H stretching vibrations, decreased in intensity and shifted slightly to lower wavenumbers, suggesting hydrogen bonding and complexation with Pb^2+^ ions. The aliphatic C–H peak at 2937 cm^− 1^ also weakened, reflecting perturbation in lipid and polysaccharide structures. The C = O stretching band of amide I at 1635 cm^− 1^ shifted and decreased in intensity, indicating the coordination of Pb^2+^ with carbonyl and amide groups. Similarly, the peaks at 1386–1251 cm^− 1^ (C–N and C–O stretching) and 1129–1083 cm^− 1^ (C–O–C in polysaccharides) showed reductions in intensity, confirming the participation of protein and carbohydrate residues in Pb^2+^ binding. The fingerprint region (845–628 cm^− 1^) exhibited additional modifications, supporting complexation between Pb^2+^ ions and organic moieties on the algal surface.

For SiO_2__2_NPs (Fig. 2d), distinct alterations were detected in the O–H and Si–OH stretching bands (3568–3480 cm^− 1^), which became less intense after adsorption, implying hydrogen bonding and surface interaction with Pb^2+^. The band at 1635 cm^− 1^ (H–O–H bending and Si–OH vibration) shifted slightly, while the strong Si–O–Si asymmetric stretching at 1050 cm^− 1^ decreased in intensity, confirming its role as a major active site. Variations in the fingerprint region (783–466 cm^− 1^) further demonstrated the incorporation of Pb^2+^ into the silica framework, likely through electrostatic attraction and surface complexation.

For the hybrid CGM–SiO_2_NPs composite (Fig. 2e), the FTIR spectrum displayed combined features of both components, indicating synergistic interactions during Pb^2+^ uptake. The broad band at ~ 3445 cm^− 1^ (O–H/N–H stretching) weakened significantly, while the C = O/Si–OH band at 1634 cm^− 1^ shifted and reduced in intensity, confirming the involvement of both carbonyl and silanol groups in Pb^2+^ coordination. The bands at 1382 cm^− 1^ (C–N/C–O) and 1173–1127 cm^− 1^ (C–O–C overlapped with Si–O–Si) also weakened, highlighting dual participation of organic and inorganic groups. Changes in the fingerprint region (834 and 626 cm^− 1^^− 1^) confirmed modifications in both C–H bending and Si–O linkages.

Overall, comparative FTIR analysis of CGM, SiO_2_NPs, and their hybrid before and after Pb^2+^ adsorption clearly demonstrates the active participation of hydroxyl, amide, carbonyl, silanol, and siloxane groups in the biosorption process. The hybrid composite exploits the functional diversity of CGM and the high surface activity of SiO_2_NPs, enabling multiple binding mechanisms such as electrostatic attraction, hydrogen bonding, and surface complexation resulting in superior Pb^2+^ removal efficiency. These observations align with previously reported FTIR findings for hybrid biosorbents used in heavy-metal remediation^31^.

The FTIR findings are further supported by the SEM observations, where the visible surface roughening, aggregation, and Pb^2+^ deposition on the biosorbent surfaces corroborate the strong interaction between Pb^2+^ ions and the functional groups (hydroxyl, carbonyl, and silanol) identified spectroscopically. This combined evidence confirms the synergistic mechanism of the CGM–SiO_2_NPs hybrid, which integrates both organic and inorganic binding sites to achieve highly efficient lead removal^32^.

### Methodology phases

**Fig. 3:**
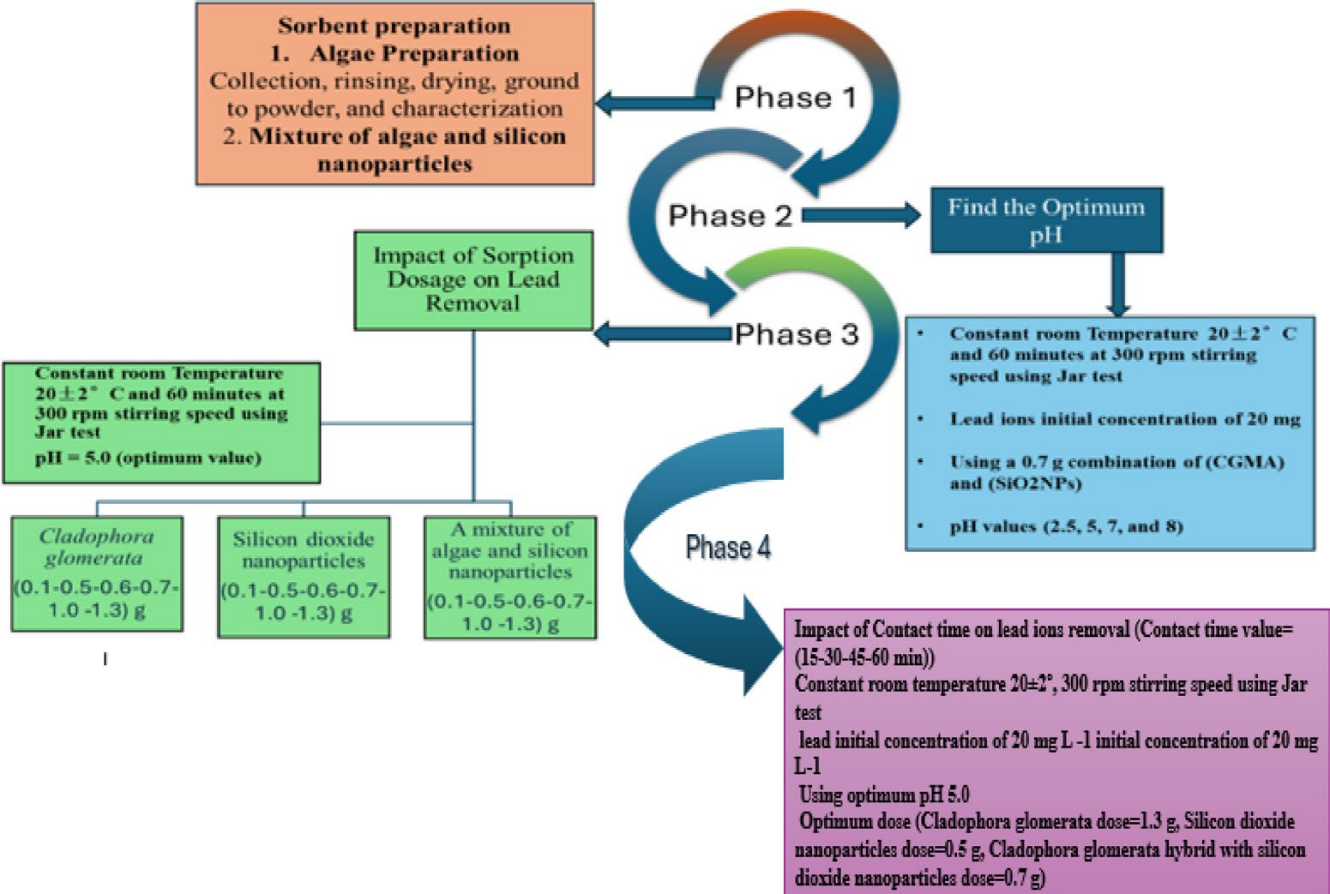
The methodology plan of the study.

The research methodology phases (shown in Fig. 3) begin with the preparation of the sorbent materials, where three types of adsorbents are tested in this study: CGM, SiO_2_NPs, and a mixture of CGM and SiO_2_NPs in a 1:1 ratio. Two key factors are evaluated to determine optimal conditions for lead adsorption: pH and adsorbent mass. The pH levels tested are 2.5, 5.0, 7.0, and 8.0 using a 0.7 g combination of CGM and SiO_2_NPs. The mass of the adsorbent used varies between 0.1 g, 0.5 g, 0.6 g, 0.7 g, 1.0 g, and 1.3 g. The experiments were conducted to investigate the adsorption of lead ions from a 200 mL solution with an initial concentration of 20.0 mg L^− 1^. All adsorption experiments were conducted at a constant room temperature of 20 ± 2 °C with a stirring speed of 300 rpm for 60 min using the Jar test.

**Fig. 4:**
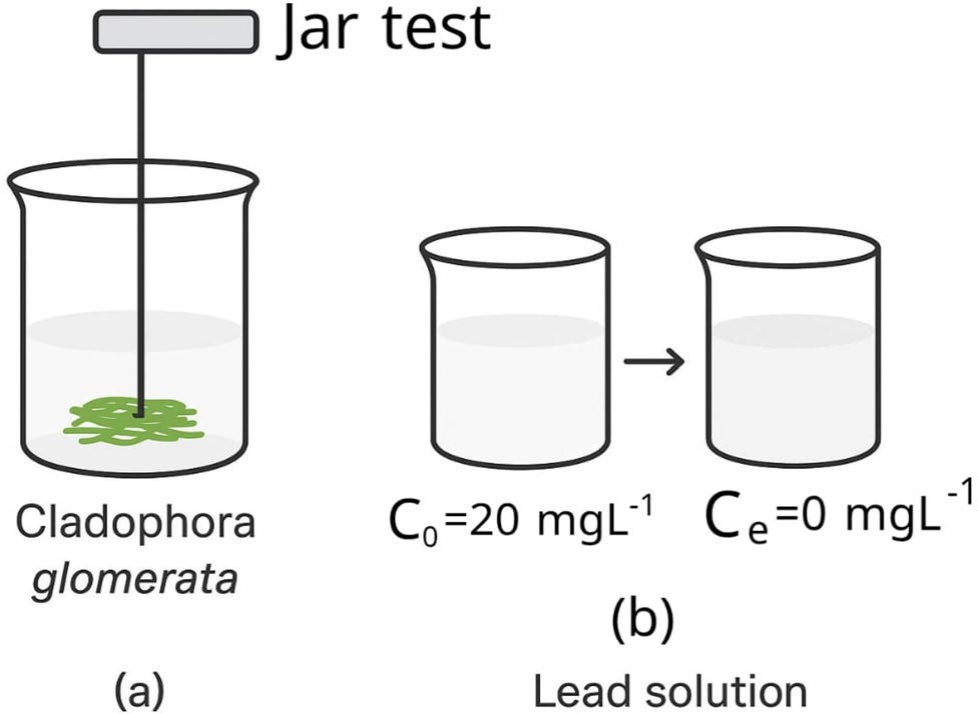
Photos of **a** Jar test during the absorption experiment using CGM and **b** Lead solution after biosorption.

Figure 4a illustrates a photo of the jar test during the absorption experiment using CGM, and Fig. 4b illustrates a photo of the lead solution after the bio-absorption.

### Validation using real industrial wastewater

To validate the adsorbent performance under realistic conditions, real industrial wastewater was collected from a local metal-finishing facility known to discharge Pb-containing effluents. The collected sample was first characterized for pH, total dissolved solids (TDS), chemical oxygen demand (COD), and native Pb concentration using atomic absorption spectroscopy (AAS). The sample was then spiked with analytical-grade Pb(NO₃)_2_ to adjust the Pb concentration to 20 mg L^− 1^, matching the conditions used in the synthetic batch experiments. The spiking volume was calculated according to:

*V*_*spike*_*=*$$\:\frac{V\:total\:x(\:C\:target-C\:real)}{C\:stock}$$ (7).

where C target = 20 mgL^− 1^, C real​ is the measured Pb concentration in the sample, V total​ is the test solution volume, and C stock​=1000 mgL^− 1^ Pb. Batch adsorption experiments were then carried out under the same optimized conditions (adsorbent dose, pH, contact time, temperature, and agitation) used for the synthetic solution tests.

## Results and discussion

### Impact of pH on lead reduction

The pH value, representing the hydrogen ion concentration, plays a crucial role in influencing the biosorption behavior of metal ions in aqueous solutions^29^. The variation in hydrogen ion concentration directly impacts the solubility and chemistry of the metal ions, as well as the availability of metal binding sites on the surfaces of biosorbents. The impact of pH on the biosorbents’ sorption of lead ions was investigated. All sorption batch experiments were conducted at a constant room temperature of 20 ± 2 °C in a 200 mL beaker with continuous swirling at 300 rpm. The experiments utilized a sorbent dose of 0.7 g of algae hybridized with silicon dioxide nanoparticles and an initial lead concentration of 20.0 mgL^− 1^.

**Fig. 5:**
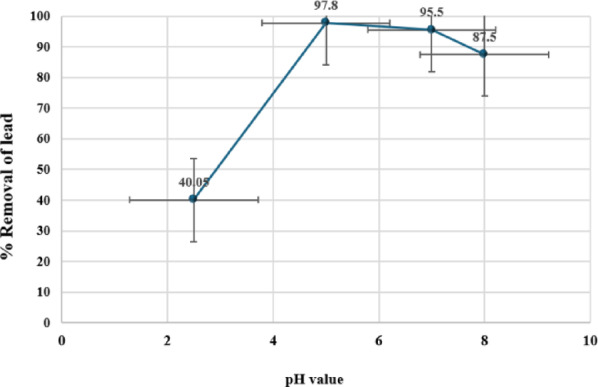
Impact of pH value on the removal of lead.

As shown in Fig. 5, the pH’s initial value significantly impacted the efficiency of the CGM hybrid with SiO_2_NPs biosorbent for lead ions removal. At a pH of 5.0, the lead ions’ sorption exhibited a significant increase, reaching its maximum value. This pH condition also resulted in a remarkable removal efficiency of 97.8% (The removal efficiency (R%) of Lead was calculated using Eq. 2). Table 2 provides the lead concentrations after the adsorption experiments at different pH values.

The reduced sorption capacity at low pH (< 3.0) can be attributed to the high concentration of hydrogen ions^30^. These hydrogen ions occupy the active binding sites, thereby inhibiting the sorption of lead ions. The slight decline in the uptake of lead ions above a pH of 5.0 can be attributed to the interference caused by sodium ions. ^30^, resulting from sodium hydroxide in the buffer solution. This prediction has been validated through a study on the effect of sodium ions as interfering ions on the sorption capacity values of lead ions^30^.

The notable rise in sorption capacity values within the pH range of 3.0–5.0 can be attributed to the investigation of the surface structure of the biosorbents. Additionally, the improvement in biosorption capacity on the surface of the solid support of silicon dioxide nanoparticles is due to the increased surface area of the biosorbent, which facilitates a greater binding of metal ions^30^. The adsorption process of heavy metal ions, such as lead ions, increases as the pH of the solution increases^33^ and then starts to decrease between pH 6.0 and 8.0 due to the precipitation process caused by the alkaline medium.

At low acidity levels, the adsorption process decreases because there is competition between heavy metal ions and H_3_O ions for the active sites in the algae. This competition reduces or even eliminates the adsorption process. Other researchers have also obtained similar results.

**Table 2:** Concentrations of lead ions after biosorption with different pH values.

pH value	2.5	5.0	7.0	8.0
Ce (mgL^− 1^) (Mean ± SD)	**11.99 ± 0.15**	**0.44 ± 0.01**	**0.90 ± 0.02**	**2.50 ± 0.05**

### The effect of the sorbent type and dosage on lead reduction

To investigate the impact of the sorbent dosage on reducing the lead concentration, a set of experiments has been conducted under optimal buffering conditions with a pH of 5.0. Various sorbent doses ranging from 0.1 g to 1.3 g were studied, along with an initial lead ion concentration of 20.0 mgL^− 1^. Sorption experiments were conducted at a constant room temperature of 20 ± 2 °C. All biosorption tests were conducted in a 200 mL beaker with constant swirling at 300 rpm for 60 min.

**Fig. 6:**
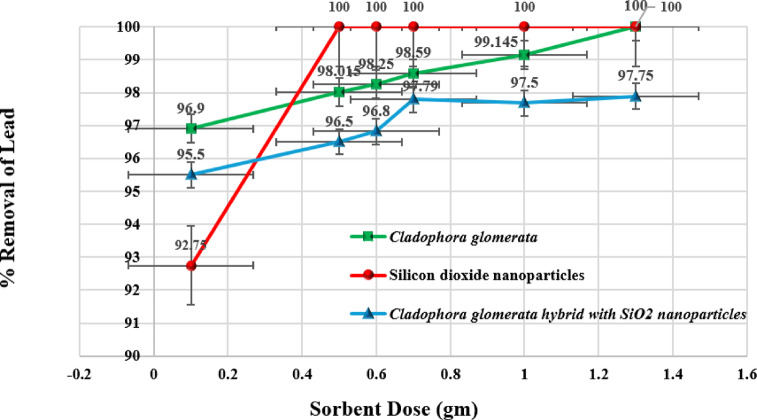
The impact of sorbent doses on the percentage of lead ions removal.

Figure 6 presents the effect of sorbent dosage on the percentage removal of lead ions. Table 3 represents the concentrations of lead and its removal efficiencies after adding different amounts of the three sorbents (CGM, SiO_2_NPs, and a mixture of CGM & SiO_2_NPs).

**Table 3:** The concentrations of lead ions with different adsorbent types and doses.

Adsorbent	C_o_ (mgL^-1^)	Removal (%) *(Mean* ± *SD)*	Ce (mgL^-1^) *(Mean* ± *SD)*	qe (mgg^-1^) *(Mean* ± *SD)*
CGM	20	95.89 ± 0.1	0.822 ± 0.02	2.95 ± 0.003
SiO_2_NPs	20	97.9 ± 0.05	0.42 ± 0.01	7.832 ± 0.0046
CGM + SiO_2_NPs	20	95.4 ± 0.1	0.92 ± 0.02	5.45 ± 0.0085

*LOD = 0.006

The results shown in Fig. 6 demonstrated that increasing the mass of CGM and SiO_2_NPs improved the adsorption percentage of lead in water. At low sorbent dosages (0.1 mgL^− 1^), CGM is superior to SiO_2_NPs in terms of lead ions reduction. However, when sorbent dosage was increased (from 0.5 mgL^− 1^), the lead reduction achieved by SiO_2_NPs was higher. The highest percentage removal of Lead achieved by CGM, SiO_2_NPs, and CGM hybrid with SiO_2_NPs was 100%, 100%, and 97.79%, respectively, at 1.3 g, 0.5 g, and 0.7 g dosage. Because of the adsorption Site Competition, CGM and SiO_2_ NP possess active sites that can adsorb lead ions. When combined, these materials might compete for the same lead ions, leading to suboptimal utilization of adsorption sites. CGM hybrid with SiO_2_NPs showed fewer increments of the percentage of adsorption on lead ions than CGM and SiO_2_NPs after 0.6 g of dosage. Increasing the sorbent dosage (CGM or SiO_2_NPs) impacts the lead ions’ reduction and may achieve complete adsorption or reach an equilibrium state. This equilibrium state is reached when the adsorption process reaches a plateau at a fixed metal concentration.

CGM has a limited number of functional groups and adsorption sites. Increasing the dosage increases the adsorption capacity, but excessive dosage may lead to particle agglomeration, reducing the available surface area. ^34^SiO_2_ NPs offer a high surface-area-to-volume ratio, but their adsorption sites may become saturated faster than CGM. The hybrid sorbent has a more accessible and interconnected network of active sites, facilitating faster diffusion of adsorbates. The heterogeneous surface chemistry of the hybrid enhances adsorption kinetics by providing multiple binding mechanisms (electrostatic interactions, hydrogen bonding, and physical adsorption)^35^ Reduced mass transfer resistance due to the nanoscale structure allows the hybrid sorbent to reach equilibrium earlier than individual CGM or SiO_2_ NPs.

The results indicate that CGM alone is effective for the reduction of lead ions. However, with these removals, CGM is not significantly different than SiO_2_NPs (paired t-test, α = 0.05). CGM is cost-effective compared to synthetic adsorbents, making it an economical and environmentally friendly choice for adsorption applications. Unlike many synthetic absorbents, algae are non-toxic and pose no significant environmental hazards, making them ideal for applications in environmentally sensitive areas. Algae was shown to perform well in treating complex wastewater with mixed contaminants. While SiO_2_NPs offer excellent performance in adsorption due to their high surface area and tunable properties, their cost is relatively high. CGM demonstrated a high removal efficiency for lead ions, even at a low dosage of 0.1 g.

Numerous research studies have been carried out for biosorption applications^36^. Focused on optimizing lead ions biosorption carried out by living cells of Arthrospira platensis. They found that when the biomass was separated from the experimental solutions by filtration, almost 50% of the initial metal dose was removed, and demonstrated that the optimum conditions for lead (II) biosorption were metal initial concentration 100 mgL^− 1^, pH 4.5, and time 60 min.

^37^ investigated the potential of Spirulina sp. for biosorption of Cd and lead ions from waste effluent. The optimum uptake for lead ions was shown at a metal concentration of 100 mgL^− 1^ was 93.6% during 15 h. The optimum adsorption of Lead was achieved at 1.5 gL^− 1^ of algal biomass, and the Optimum removal showed at an agitation rate of 150 rpm was 89.9%. Both metals showed optimum adsorption at 1.5 gL^− 1^ of algal biomass and lead ions by 89.2%.

^38^ Investigated the removal capacity of lead ions using the biomass of dried cattle manure in an aqueous solution. The adsorption rate was rapid, reaching equilibrium after 25 min and removal of 96.8%.

### The impact of the sorbent type and dosage on the sorption capacity

**Fig. 7:**
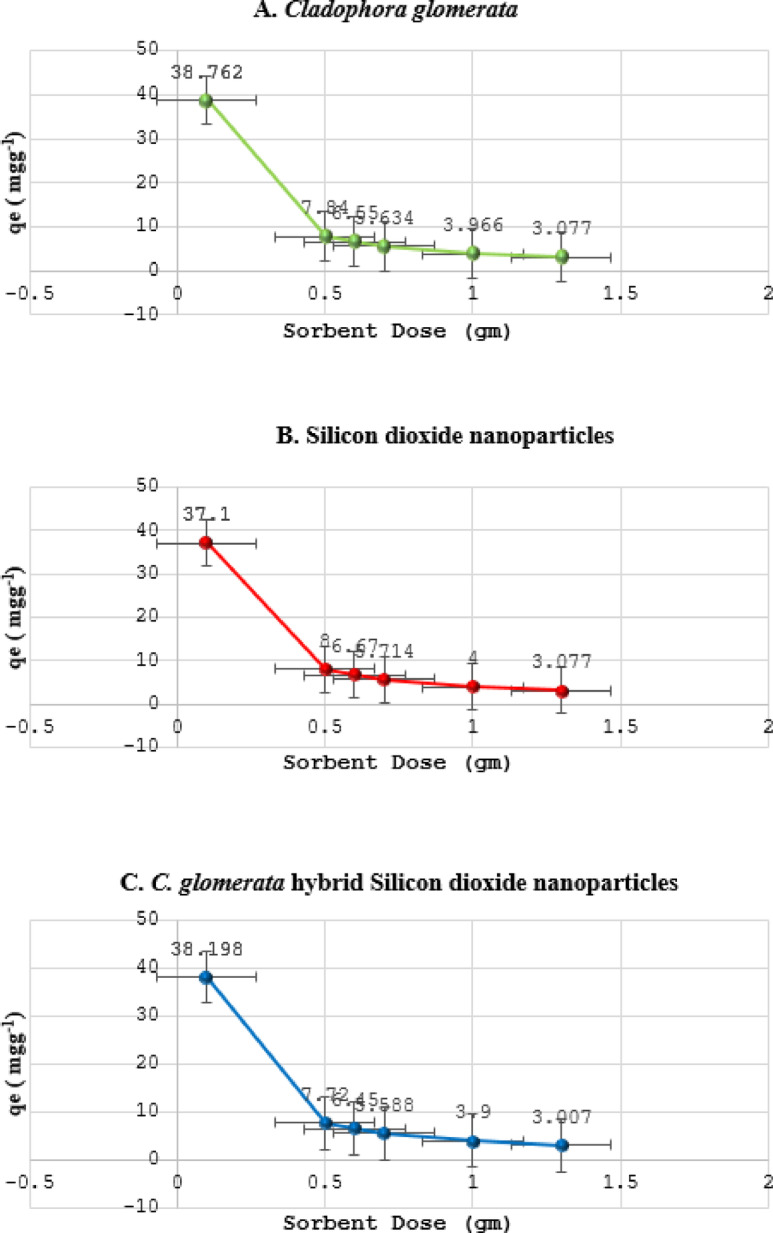
The effect of adsorbent dosage on the Sorption capacity of the Lead ions adsorption: (A) C. glomerata, (B) Silicon dioxide nanoparticles, and (C) C. glomerata hybrid Silicon dioxide nanoparticles.

The impact of sorbent type and dosages on the sorption capacity (calculated using Eq. 3) was also investigated and presented in Fig. 7. Sorption capacity values were investigated under the optimum condition of pH 5.0 by using different sorbent doses (0.1–1.3 g).

The adsorption values decreased from 38.762 mg g–1 when using 0.1 g of algae to 3.077 mg g–1 when using 1.3 g of biomass. Similarly, the values decreased from 37.1 mg g^– 1^ when using 0.1 g of SiO_2_NPs to 3.077 mg g^– 1^ when using 1.3 g. The values decreased from 38.198 mg g^– 1^ when using 0.1 g of CGM hybrid with SiO_2_NPs to 3.012 mg g^–1^ when using 1.3 g. The percentage of lead removal increased when the sorbent dose was amplified.

The results indicate that with an increase in the adsorbent dosage, the number of available binding sites and outer layer patches for ions to bind also increases^38^. Once all the available binding sites are occupied by lead ions, increasing the dosage of adsorbent may result in diminishing returns or even a decrease in the amount of lead ions adsorbed per unit mass of adsorbent^30^. This is because the excess adsorbent does not provide additional binding capacity and instead occupies space without contributing to further adsorption^30^. While initially increasing the dosage of adsorbent enhances the number of available binding sites and outer layer patches for ions to bind to, there is a point where further increases in dosage do not result in proportional increases in lead ions’ adsorption capacity. This is typically due to the saturation of binding sites and the presence of excess adsorbent that does not effectively contribute to the adsorption process^30^.

The same results were revealed from previous studies^38^. Investigated the removal of lead ions using the biomass of dried cattle manure with doses (0.1, 0.15, 0.2, and 0.25 g) from the aqueous solution. The study was assessed at pH 7.5, 200 rpm, temperature 18 °C, at 30 mg·dm–3 of lead ions for 35 min. It has been noted that when the dose increased from 0.1 to 0.25 g, the adsorption capacity of lead ions decreased from 13.256 to 5.919 mg·g^− 1^, which could be due to the low availability of lead ions to fill the active sites of the bio-adsorbent. Another study was conducted by^39^ To investigate the impact of the adsorbent dose on the removal of lead ions present in a synthetic solution using sugarcane bagasse (Saccharum officinarum). As the adsorbent dose increased from 0.05 to 0.1 g, the adsorption capacity decreased from 24.04 to 12.46 mg. g^− 1^. It was reported that the surface area and availability of active sites of the biomaterial are proportional to the amount of adsorbent in contact with the contaminated solution. However, with a high amount of adsorbent, the available lead ions are insufficient to cover all adsorption sites, and the agglomeration of active sites results in low metal adsorption.

### Impact of contact time on lead reduction

**Fig. 8:**
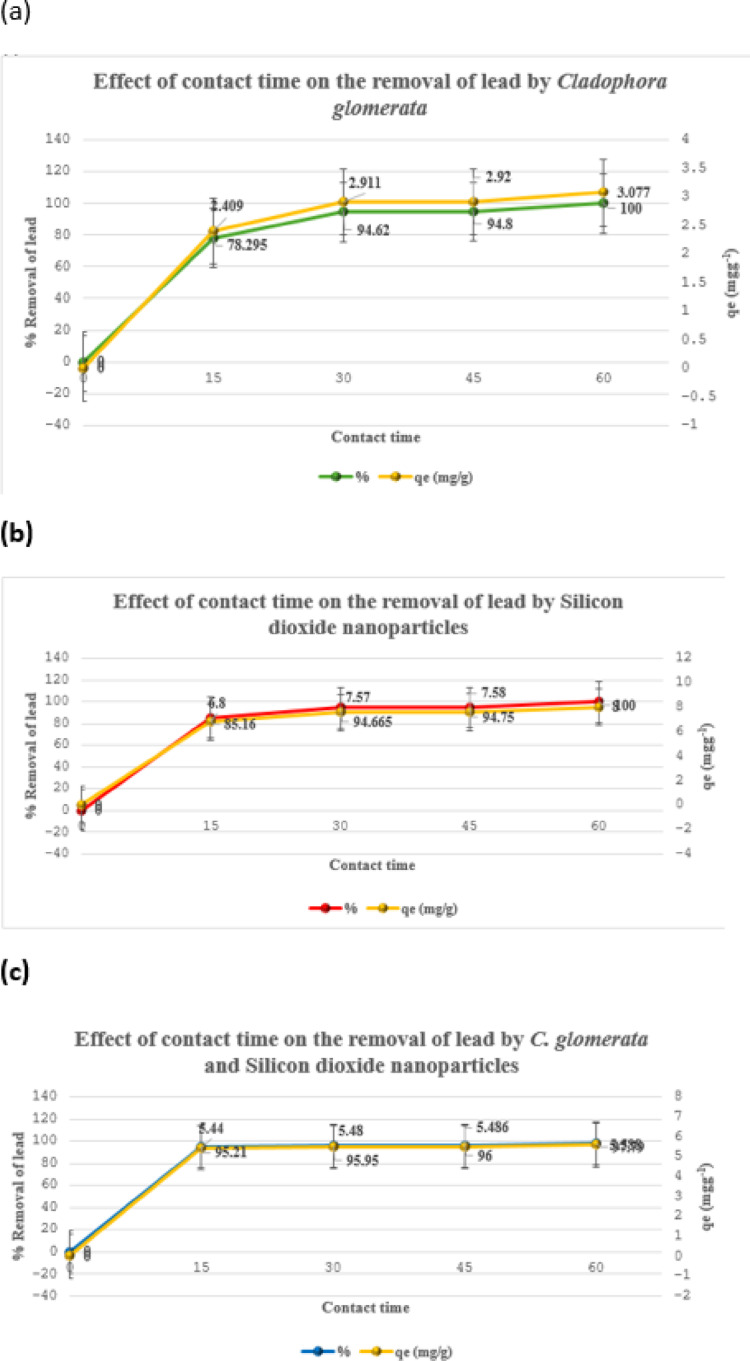
The Effect of contact time on the removal of lead ions by **a** CGM at ideal dosage = 1.3 g, **b** SiO_2_ NP at ideal dose = 0.5 g, and **c** CGM hybrid with SiO2 nanoparticles at ideal dose = 0.7 g.

Figure 8 illustrates the effect of contact time on the removal efficiency of lead and the adsorption capacity (qe​) using different sorbents. The percentage removal of lead increases rapidly from 0% at the beginning to about 78.295% within the first 15 min using CGM, as shown in Fig. 8(a). It then increases gradually and reaches 100% at 60 min. The adsorption capacity (qe) also increases quickly from 0 mgg^− 1^ at the start to 2.409 mgg^− 1^ within the first 15 min. Figure 8 (b) illustrates the effect of contact time on the removal percentage of lead and the adsorption capacity (qe) using SiO2 NP. The percentage of lead removed increases quickly, from 0% at the beginning to about 85.16% within the first 15 min. After 15 min, the removal percentage continues to increase gradually and stabilizes around 100% as the contact time reaches 60 min. The adsorption capacity (qe) also increases rapidly from 0 mgg^− 1^ at the start to 6.8 mgg^− 1^ within the first 15 min. Figure 8 (c) shows the effect of contact time on the adsorption of lead using the CGM hybrid with SiO2 NP. In the beginning (0 to 15 min), there is a rapid increase in the percentage removal of lead. The percentage removal reaches 95.21%, and the adsorption capacity reaches about 5.44 mgg^− 1^. The results indicate that the majority of lead adsorption by the sorbents occurred within the first 15 min of contact. During this initial phase, the adsorption sites were highly accessible, facilitating rapid interaction with lead ions and resulting in a sharp increase in both percentage removal and adsorption capacity. Beyond 15 min, the adsorption rate significantly declined, suggesting that equilibrium had been reached, and most active sites were occupied. Minor fluctuations observed after equilibrium may be attributed to desorption and re-adsorption. Table 4 presents the lead concentrations measured at various contact times following biosorption using the optimal dose of each sorbent.

**Table 4:** The concentrations of lead with different contact times and doses.

Contact time (min)	CGM			SiO_2_NPs			CGM & SiO_2_NPs		
	lead Ce (mgL^− 1^) (Mean ±SD)	Lead Removal (%) (Mean ± SD)	qe (mgg^− 1^) (Mean ± SD)	lead Ce (mgL^− 1^) (Mean ± SD)	lead Removal (%) (Mean ± SD)	qe (mgg^− 1^) (Mean ± SD)	lead Ce (mgL^− 1^) (Mean ± SD)	lead Removal (%) (Mean ± SD)	qe (mgg-^1^) (Mean ± SD)
15	4.34 ± 0.01	78.29 ± 0.044	2.41 ± 0.001	2.97 ± 0.03	85.16 ± 0.1	6.80 ± 0.02	0.96 ± 0.011	95.21 ± 0.055	5.44 ± 0.003
30	1.08 ± 0.001	94.62 ± 0.007	2.91 ± 0.0	1.07 ± 0.01	94.67 ± 0.05	7.57 ± 0.005	0.81 ± 0.007	95.95 ± 0.034	5.48 ± 0.0025
45	1.03 ± 0.015	94.80 ± 0.07	2.92 ± 0.002	1.05 ± 0.01	94.75 ± 0.05	7.58 ± 0.004	0.8.00 ± 0.01	96.00 ± 0.05	5.49 ± 0.0025
60	0.00 ± 0.0	100 ± 0.0	3.08 ± 0.0	0.00 ± 0.0	100 ± 0.0	8.00 ± 0.0	0.44 ± 0.005	97.79 ± 0.026	5.59 ± 0.001

### Comparison of adsorption performance and mechanism

The superior adsorption performance of the prepared materials can be explained by their structural and surface characteristics. SiO_2_ nanoparticles inherently provide a high surface-area-to-volume ratio, resulting in numerous active sites for Pb^2+^ binding; however, the limited chemical diversity of functional groups causes these sites to saturate more rapidly compared to CGMA biomass. In contrast, CGMA features abundant hydroxyl, carboxyl, and amino moieties, promoting strong complexation and electrostatic attraction with Pb^2+^ ions, which accounts for its high removal efficiency even at a low dosage of 0.1 g. Incorporating SiO_2_ NPs into a hybrid biosorbent matrix (CGMA) further reduces intraparticle mass transfer resistance by enhancing porosity and dispersing nanoparticles on the fibrous framework, thus accelerating adsorption kinetics and increasing capacity. Similar synergistic enhancements and mechanistic explanations for biosorbent–nanomaterial composites have been reported in the literature^40^ .

### Comparative adsorption performance in synthetic and real wastewater

The real wastewater sample was characterized (pH, TDS, COD, native Pb^2+^ concentration) and spiked to 20 mg L^− 1^ Pb^2+^ prior to adsorption tests, following the procedure described in Sect. 2.7.

Batch adsorption experiments were then performed under the same optimized conditions previously determined for the synthetic solution tests (pH 5.0, adsorbent dose of 1.3 g CGM / 0.5 g SiO_2_ NPs / 0.7 g hybrid, contact time 60 min, temperature 20 ± 2 °C, and agitation speed 300 rpm).

As presented in Table 4, all adsorbents demonstrated high Pb^2+^ removal efficiencies in real wastewater, with slightly lower performance than in synthetic solutions. CGM achieved ~ 96% removal, SiO_2_ NPs achieved ~ 98%, and the hybrid adsorbent exhibited 95.4% removal. The slight decrease in performance can be attributed to competitive adsorption from coexisting cations (Ca^2+^, Mg^2+^, Na⁺) and the presence of dissolved organic matter that may block or compete for active binding sites.

These findings are consistent with previous reports, which have noted similar reductions in removal efficiency under complex wastewater matrices. The slight decrease in performance can be attributed to the presence of competing ions in the industrial matrix, which reduces Pb^2+^ uptake, consistent with findings by^41^ on competitive adsorption behavior. Additionally, the presence of dissolved organic matter may block or occupy active adsorption sites, reducing capacity a mechanism highlighted in recent reviews of adsorption in complex wastewater systems^42^.

The adsorption results for the real industrial wastewater are summarized in Table 5, which presents the removal efficiencies of Pb^2+^ using CGM, SiO_2_ NPs, and the hybrid adsorbent under optimized conditions.

**Table 5:** Adsorption results for real industrial Wastewater.

Adsorbent	C_o_ (mgL^− 1^)	Removal (%) (Mean ± SD)	Ce (mgL^− 1^) (Mean ± SD)	qe (mgg^− 1^) (Mean ± SD)
CGM	20	95.89 ± 0.1	0.822 ± 0.02	2.95 ± 0.003
SiO_2_NPs	20	97.9 ± 0.05	0.42 ± 0.01	7.832 ± 0.0046
CGM + SiO_2_NPs	20	95.4 ± 0.1	0.92 ± 0.02	5.45 ± 0.0085

### Validation of adsorbent performance using real Wastewater — Literature perspective

While the present study focused on synthetic Pb^2+^ solutions to elucidate adsorption mechanisms, several studies underscore the need to validate materials under real effluent conditions. For example, Noug stalk activated carbon removed 94.84% of Pb^2+^ from paint factory wastewater even with high COD (1717 mgL^− 1^) and TDS (1231 mgL^− 1^) confirming biosorption viability in complex matrices^43^. Similarly, a biosorbent derived from *Tinospora cordifolia* demonstrated notably effective removal from lead–acid battery effluent. ^44^ Moreover, an activated carbon (Carbon C) achieved 97.86% Pb^2+^ removal with a maximum capacity of 48.75 mgg^− 1^ in industrial wastewater samples^45^. Reviews of Pb^2+^ biochar adsorption consistently report high removal efficiencies (≥ 80–90%) even in complex matrices highlighting biosorption’s practical potential^46^. Taken together, these findings suggest that while matrix interferences may slightly decrease performance, biosorbents can still achieve robust removal in actual wastewater. Accordingly, future work on CGM–SiO_2_ NPs should include validation using spiked or real effluents, applying standardized characterization and spiking protocols to substantiate practical applicability.

### Kinetic modeling analysis

#### Pseudo‑First order

**Fig. 9:**
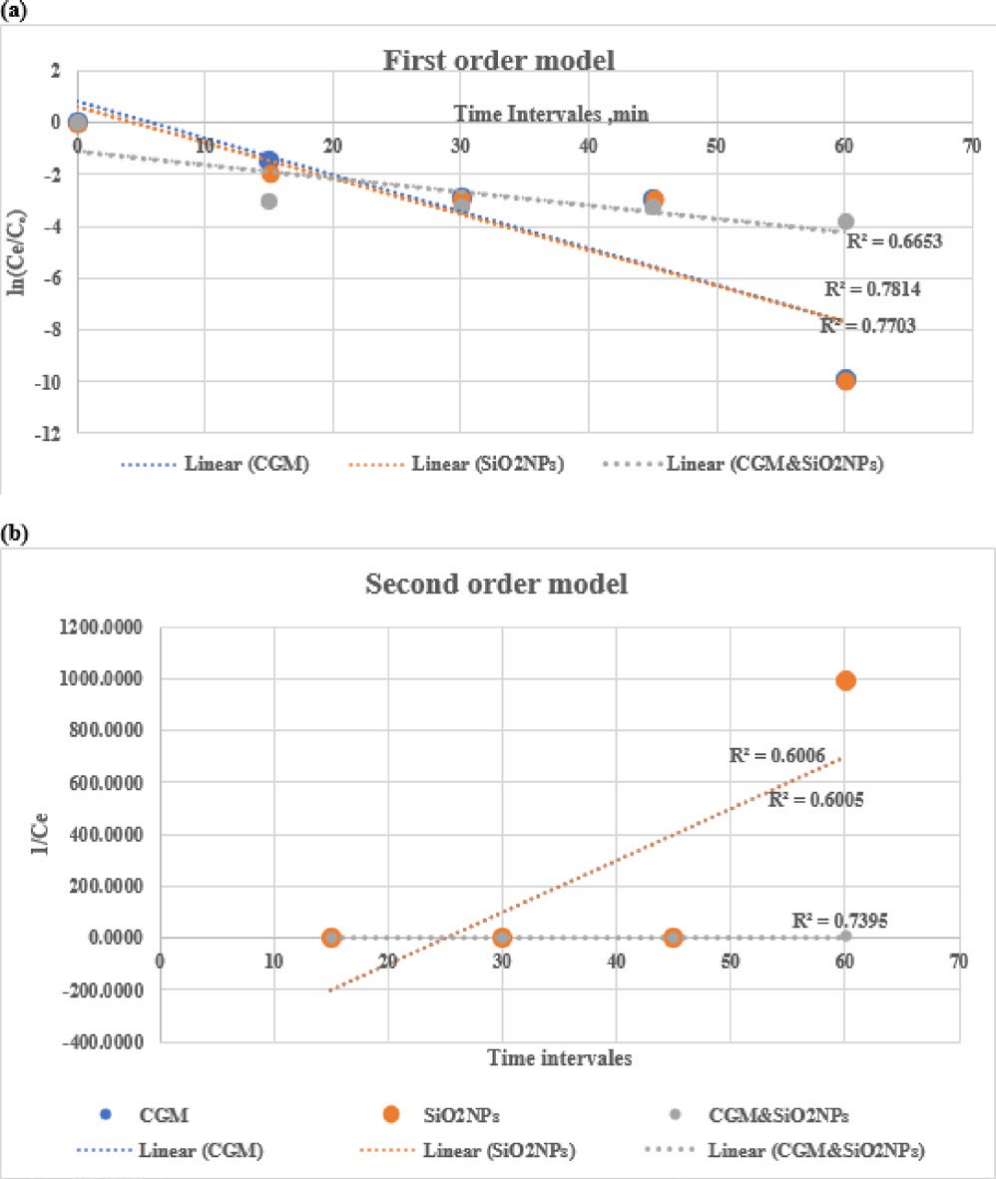
**a** Pseudo-first order graph; **b** pseudo-second-order graph for the biosorption of lead ions onto CGM, SiO2NPs, and CGM&SiO2NPs.

Figure 9a presents the plot of ln(C/C_0_) versus time (t), which is used to evaluate the pseudo-first-order kinetic model. The calculated rate constant (K_1_) and the corresponding linear regression correlation coefficient (R_1_^2^) values are summarized in Table 6.

#### Pseudo‑second order

The second-order rate constant (K_2_) was determined from the slope of the plot of ***1/C*** versus time ***(t)***, as shown in Fig. 9***(b)***. Table 6 presents the calculated K_2_ values along with their corresponding correlation coefficients ***(R***_***2***_***²)***.

**Table 6:** Kinetic constants for lead ions biosorption onto CGM, SiO_2_NPs, and CGM–SiO_2_NPs composites at an initial concentration (C_0_) of 20 mg L^− 1^.

Sorbent	K_1_ (min^− 1^)	$\:{\varvec{R}}_{1}^{2}$	K_2_ (L mg^− 1^ s^− 1^)	$\:{\varvec{R}}_{2}^{2}$
CGM	0.086	0.781	0.056	0.601
SiO2NPs	0.091	0.770	0.056	0.601
CGM & SiO_2_NPs	0.089	0.665	0.001	0.739

The chart in Fig. 9 (a) shows the first-order model, illustrating the adsorption kinetics of lead ions using different adsorbents over time. The y-axis represents the natural logarithm of the concentration ratio, ln (Ce/C₀), indicating how the concentration of the contaminant in solution changes relative to its initial concentration over time (x-axis, in minutes). Three different adsorbent systems are compared: **CGM** (blue dotted line), SiO_2_NPs (orange dotted line), and CGM & SiO_2_NPs composite (gray dotted line). Each dataset is fitted with a linear regression line, with the corresponding R² values indicating the goodness of fit for the first-order kinetic model: **CGM**: R² = 0.7814, SiO_2__2_NPs: R² = 0.7703, and CGM & SiO_2_NPs: R² = 0.6653. The plot demonstrates that both CGM and SiO_2_NPs follow first-order adsorption kinetics reasonably well, with R² values of 0.7814 and 0.7703, respectively, suggesting a good correlation between experimental data and the first-order model. In contrast, the composite system (CGM & SiO_2_NPs) exhibits a lower R² value (0.6653), indicating a weaker fit to the first-order model. These results suggest that while the individual materials exhibit first-order adsorption behavior, their combination may involve more complex kinetic interactions, potentially requiring a different kinetic model.

The adsorption kinetics of CGM, SiO_2_NPs, and their combination (CGM & SiO_2_NPs) were evaluated using the second-order kinetic model, as presented in Fig. 9**(b)**. The linearized form of the second-order model was applied by plotting (1/Ce) versus time for each adsorbent system. The corresponding R² values were derived to assess the goodness of fit for each dataset. From the figure, it is evident that the *CGM & SiO_2__2_NPs* system exhibits the highest correlation coefficient (R² = 0.7395), indicating a better fit to the second-order model compared to CGM (R² = 0.6006) and SiO_2_NPs (R² = 0.6005) individually. This suggests that the combined use of CGM and SiO_2_NPs results in more effective adsorption kinetics. In contrast, the individual systems (CGM and SiO_2_NPs) show moderate linearity with similar R² values, reflecting that while each material demonstrates adsorption capability, their performance in fitting the second-order model is less robust compared to the composite system.

Notably, the linear trend of the SiO_2_NPs data is characterized by a steeper slope, which may indicate a more dynamic change in equilibrium concentration over time. On the other hand, the CGM & SiO_2_NPs trend line is relatively flat, suggesting a more gradual and possibly controlled adsorption process.

The hybrid biosorbent demonstrated improved removal efficiency and faster kinetics compared to its individual components, confirming the synergistic effect of integrating SiO_2_ nanoparticles with algal biomass.

These findings support the hypothesis that combining CGM with SiO_2_NPs enhances the adsorption efficiency and kinetics, likely due to increased active surface sites and improved diffusion pathways.

#### Pseudo-Second Order-Non-Linear

**Fig. 10:**
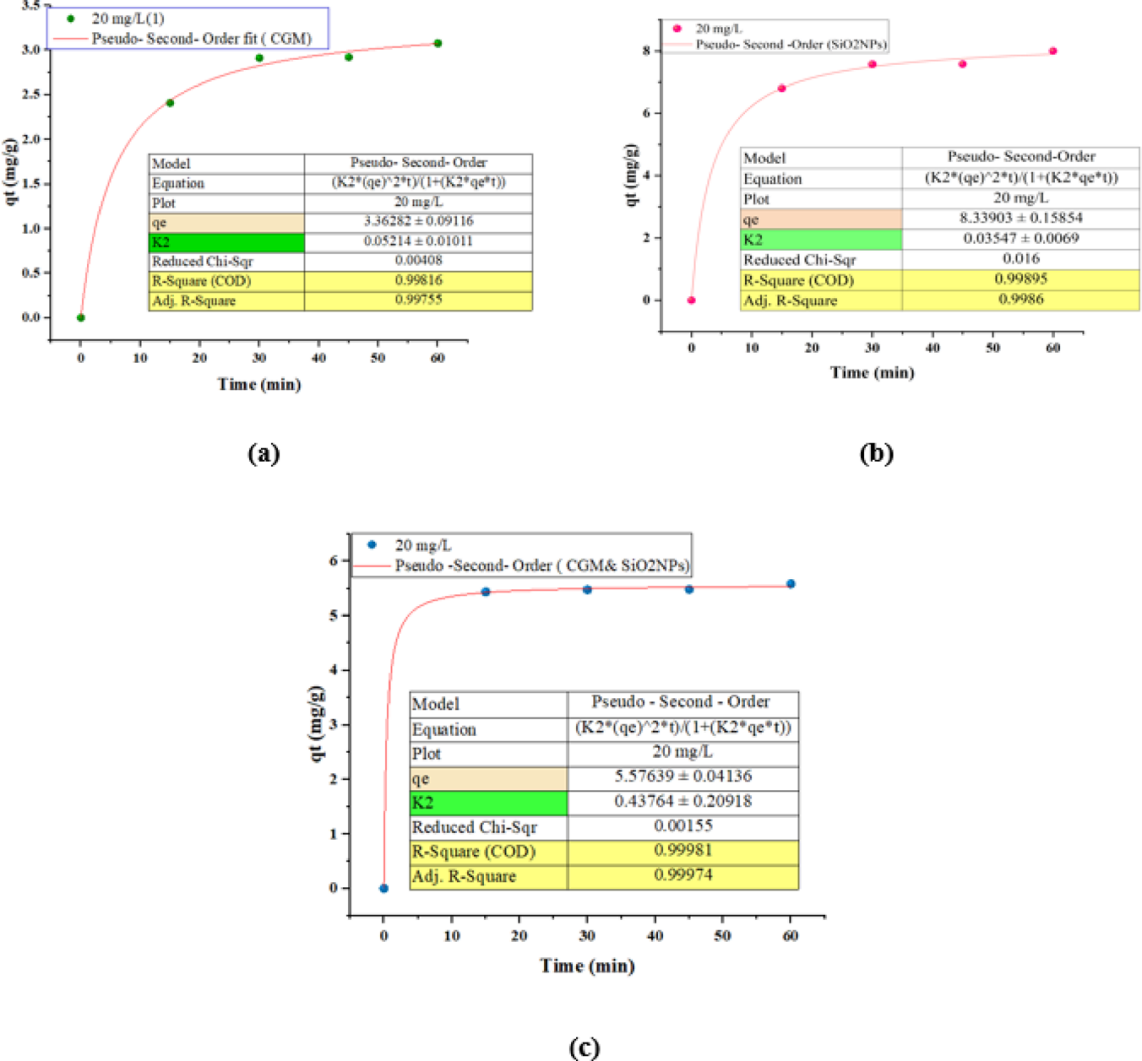
Pseudo-second-order nonlinear model for fitting the experimental data of CGM, SiO2NPs, CGM & SiO2NPs.

Figure 10**(a)** presents a kinetic adsorption plot showing the fit of experimental data to the Pseudo-Second Order (PSO) kinetic model for an adsorbate concentration of 20 mgL^− 1^. The plot illustrates how the adsorption capacity, qt​ (mgg^− 1^), varies with contact time (min). The data (green dots) are fitted using a nonlinear form of the PSO equation. Equilibrium adsorption capacity (q_e_): 3.36282 ± 0.09116 mgg^− 1^, rate constant (K_2_): 0.05214 ± 0.01011. These values indicate an **excellent fit** of the experimental data to the PSO model, implying that **chemisorption** may be the dominant mechanism controlling the adsorption kinetics. The adsorption kinetics of the target contaminant at an initial concentration of 20 mgL^− 1^ were best described by the pseudo-second-order model, as evidenced by a high correlation coefficient (R² = 0.99816) and a low reduced Chi-square value (0.00408). Figure 10**(b)** presents adsorption kinetics of the same 20 mgL^− 1^ solution but now using SiO_2_ nanoparticles (SiO_2_NPs) as the adsorbent. The data were again fitted with the **Pseudo-Second Order (PSO)** kinetic model, as indicated by the high-quality curve fitting and the tabulated statistical parameters. Equilibrium adsorption capacity (q_e_): 8.33903 ± 0.15854 mgg^− 1^, Rate constant (K_2_): 0.03547 ± 0.0069. Compared to the previous case (without SiO_2_NPs), the use of SiO_2_NPs more than doubled the adsorption capacity (q_e_), indicating enhanced adsorption performance, possibly due to increased surface area, active sites, or surface chemistry of the nanoparticles. When SiO_2_ nanoparticles were employed as the adsorbent at the same initial concentration (20 mgL^− 1^), the pseudo-second-order kinetic model still provided an excellent fit (R^2^ = 0.99895, Adjusted R² = 0.9986), affirming the dominance of chemisorption mechanisms. The equilibrium adsorption capacity increased significantly to 8.34 mgg^− 1^, suggesting that SiO_2__2_NPs substantially enhance the adsorption performance compared to the conventional material. This improvement can be attributed to the higher surface area and availability of active sites provided by the nanoparticles. Figure 10**(c)** represents the adsorption kinetics for a 20 mgL^− 1^ solution using a composite adsorbent of CGM and SiO_2_ nanoparticles. The kinetic behavior was once again modeled using the **Pseudo-Second Order (PSO)** equation, with excellent fitting as indicated by statistical measures. Equilibrium adsorption capacity (q_e_): 5.57639 ± 0.04136 mgg^− 1^, Rate constant (K_2_): 0.43764 ± 0.20918. This composite material shows an intermediate adsorption capacity between CGM alone (3.36 mgg^− 1^) and SiO_2_NPs alone (8.34 mgg^− 1^), but with a significantly higher rate constant (K_2_). This indicates **faster kinetics**, suggesting that the synergy between CGM and SiO_2_NPs may facilitate more efficient adsorption over a shorter period. The composite adsorbent comprising CGM and SiO_2_ nanoparticles also conformed exceptionally well to the pseudo-second-order model (R² = 0.99981), underscoring chemisorption as the dominant mechanism. While the equilibrium adsorption capacity (5.58 mgg^− 1^) was lower than that of pure SiO_2__2_NPs, the rate constant (K_2_ = 0.43764 g/mg·min) was substantially higher than both individual components. This suggests that the composite system not only retains significant adsorption capacity but also enhances the adsorption rate, likely due to synergistic effects such as improved dispersion of SiO_2_NPs and enhanced surface accessibility provided by the CGM.

## Environmental and practical implications

The fate of the lead-loaded adsorbents is a critical consideration for the practical application of biosorption technologies. Following adsorption, *Cladophora glomerata* biomass and SiO_2_ nanoparticles can be regenerated using appropriate desorption agents such as 0.1 M HCl, dilute NaOH, or chelating agents like EDTA, which effectively release adsorbed Pb^2+^ ions and restore adsorption capacity for multiple cycles ^47,48^.

Regeneration not only reduces operational costs but also minimizes solid waste generation. Previous studies have demonstrated that biomass- and silica-based sorbents can retain a high proportion of their adsorption performance after regeneration, although efficiency losses may occur due to partial saturation of active sites^49^.

In cases where regeneration is not feasible, safe disposal of lead-loaded adsorbents is essential to prevent secondary pollution. Immobilization techniques, such as encapsulation in cement matrices or solidification/stabilization, are widely recommended for toxic metal-laden sorbents to prevent leaching into the environment^50^.

Moreover, spent biosorbent residues (e.g., rice husk containing adsorbed Pb^2+^) have successfully been incorporated into ceramic bricks (10% by volume), where leaching tests confirmed effective immobilization of lead within the ceramic matrix, offering a sustainable disposal route^51^.

### Reusability and operational stability of the adsorbents

To evaluate the feasibility of reuse and operational stability of the prepared biosorbents, a preliminary regeneration experiment was performed for each sorbent individually, including CGM, SiO_2_NPs, and their composite (CGM + SiO_2__2_NPs). For every test, 1.0 g of each adsorbent (independently) was contacted with 200 mL of Pb^2+^ solution (20 mg L^− 1^) during the first adsorption cycle. After completion of the adsorption test, the Pb^2+^-loaded adsorbents were separated and subjected to desorption using a regenerating solution composed of hydrochloric acid, ethanol, and distilled water in a 1:1:2 volume ratio.

The saturated adsorbents were agitated in the regenerating solution at 300 rpm for 2 h using a Jar Test apparatus to ensure efficient desorption without structural deterioration. The regenerated materials were then thoroughly washed with distilled water, dried, and reused under the same operating conditions applied in the initial adsorption experiments (adsorbent dose = 1.0 g, solution volume = 200 mL, contact time = 60 min, agitation speed = 300 rpm).

A slight decrease in removal efficiency after regeneration was observed, attributed to partial saturation or irreversible binding of some active sites. However, the performance remained high, particularly for SiO_2_NPs and the composite, highlighting their promising reusability and potential cost-effectiveness for real wastewater treatment applications^52^.

As shown in Table 7, SiO_2_NPs maintained the highest regeneration efficiency (96.3%), followed by CGM (95.1%) and the hybrid (93.2%), indicating minimal performance loss after the first regeneration cycle.

**Table 7:** Regeneration performance of biosorbents for Pb^2+^ removal after the first adsorption cycle.

Adsorbent	Removal (%) (Mean ± SD)	Ce (mg L^-1^) (Mean ± SD)	qe (mg g^-1^) (Mean ± SD)
CGM	95.10 ± 0.15	0.98 ± 0.03	3.80 ± 0.002
SiO_2_NPs	96.30 ± 0.11	0.74 ± 0.02	3.85 ± 0.005
CGM + SiO2NPs	93.22 ± 0.05	1.36 ± 0.01	3.73 ± 0.002

## Limitations and future work

In the present study, the main focus was placed on understanding the biosorption kinetics of Pb^2+^ onto CGM, SiO_2_ NPs, and their hybrid, which is a critical step in evaluating adsorption efficiency and mass transfer behavior. Although adsorption isotherms and thermodynamic parameters were not investigated, they represent essential tools to fully describe the sorption mechanism, surface heterogeneity, and spontaneity of the process. Therefore, future research should include comprehensive equilibrium and thermodynamic studies under varying initial Pb^2+^ concentrations and temperatures to provide a complete understanding of the adsorption system and to optimize large-scale applications.

## Conclusion

This study demonstrated that *Cladophora glomerata* biomass (CGM), SiO_2_ nanoparticles, and their hybrid effectively removed Pb^2+^ ions from aqueous solutions and real industrial wastewater. CGM showed high efficiency even at low dosage (0.1 g), while the hybrid reduced mass transfer resistance and enhanced adsorption kinetics. The adsorption process followed the pseudo-second-order model, indicating chemisorption as the rate-limiting step. Owing to its low cost, renewability, and sustainability, CGM represents a promising biosorbent for practical wastewater treatment applications.

The developed CGM–SiO_2__2_NPs hybrid provides a novel, cost-effective, and environmentally sustainable solution for heavy-metal removal, with demonstrated applicability in real wastewater systems.

Future studies should focus on regeneration and testing against other heavy metals to ensure safe large-scale implementation.

## Supplementary Information

Below is the link to the electronic supplementary material.

Supplementary Material 1

## Data Availability

All data listed or discussed during this work are included in this published article and its supplementary information files.
