# Supplementary material for: Lead biosorption from industrial wastewater using Cladophora
Source: Sci Rep. 2025 Dec 11;15:43653. doi: 10.1038/s41598-025-29920-4 (PMC12700987; doi:10.1038/s41598-025-29920-4)
Supplement: Supplementary file 1 — Supplementary Material 1 [file 41598_2025_29920_MOESM1_ESM.docx]

**Supplementary Information for Biosorption of Pb²⁺ Using CGM–SiO₂ Nanoparticles Hybrid System**

**Molecular Identification of Algal Biomass**

Figure S1.a: Specific genomic product for 18S rRNA of isolate and Negative control with ≈ 744 bp.

Figure S1.b: Computerized detection of specific genomic product for 18S rRNA of isolate and Negative control with ≈ 744 bp.

Figure S1.c: Computerized fragments length detection specific genomic product for 18S rRNA of isolate and Negative control with ≈ 744 bp.

Figure S1.d: data analysis for DNA ladder.

Figure S1.e: data analysis for sample.

Table S1.a: data parameters for DNA ladder and samples.

Table S1.b: Specific Primer sequence under study.

| Gene | Sequences | Target bp | References |
| --- | --- | --- | --- |
| 18S rRNA | C-2**:** 5΄- ATTGGAGGGCAAGTCTGGT -3΄  D-2: 5΄- ACTAAGAACGGCCATGCAC -3΄ | 744 | Gomaa et al., 2019^1^ |

Table S1.c: Master Mix component for PCR reactions

| **Master Mix component** | **Amount** | **Final concentration** |
| --- | --- | --- |
| Sterile nuclease free water | 23 µl |  |
| **Green taq (DreamTaq) master mix** | 25 µl | 1.0 x |
| Primer (5pmol /µl) | 1.0 µl | 5.0 pmol |
| DNA extracted sample (50ng /µl) | 1.0 µl | 25.0 ng |
| Total | 50.0 µl |  |

Figure S1.f: Sample sequence

Figure S1.g: Alignment data

Figure S1.h: Sequences producing significant alignments

Table S1.d: identification data.

| **Identity %** | **Highly similarity isolate** | **GenBank** |
| --- | --- | --- |
| 94.70% | *Cladophora glomerata 3-CL-2019*  *gene for 18S rRNA, partial sequence* | LC536845.1 |

## SEM Images Before Pb²⁺ Adsorption

**Figure S2. a1 (CGM)**

SEM micrograph (×1500) showing the surface morphology before Pb²⁺ adsorption.

**Figure S2. b1 (Hybrid CGM–SiO₂NPs)**

SEM micrograph (×9500) showing the surface morphology before Pb²⁺ adsorption.

## SEM Images After Pb²⁺ Adsorption

### Figure S3. a1 (CGM)

SEM micrograph (×100) showing morphological changes after Pb²⁺ adsorption.

**Figure S3. b1 (Hybrid CGM–SiO₂NPs)**

SEM micrograph (×3700) showing morphological changes after Pb²⁺ adsorption.

## FTIR Spectra Before Pb²⁺ Adsorption

Table 4S. a. FTIR peak data for CGM (before Pb²⁺ adsorption)

Table S4. b FTIR peak data for SiO₂ nanoparticles (before Pb²⁺ adsorption)

## FTIR Spectra After Pb²⁺ Adsorption

Table S5. c FTIR peak data for CGM (after Pb²⁺ adsorption)

Table S5. d FTIR peak data for SiO₂NPs (after Pb²⁺ adsorption).

Table S5. e FTIR peak data for (Hybrid CGM–SiO₂NPs) (after Pb²⁺ adsorption).

**Reference**

1.	Gomaa, M. A., Refaat, M. H., Salim, T. M., El-Sayed, A. E. K. B. & Bekhit, M. M. Identification of green alga chlorella vulgaris isolated from freshwater and improvement biodiesel productivity via UV irradiation. *Microbiology and Biotechnology Letters* 47, 381–389 (2019).
